# Real-World Management and Survival Outcomes in Patients with Primary or Recurrent Retroperitoneal Liposarcoma: A Single-Center Retrospective Cohort Study

**DOI:** 10.3390/cancers18203334

**Published:** 2026-10-15

**Authors:** Atsuhiro Kijima, Nobuki Furubayashi, Takahito Negishi, Motonobu Nakamura

**Affiliations:** Department of Urology, National Hospital Organization Kyushu Cancer Center, Notame 3-1-1, Minami-ku, Fukuoka 811-1395, Japan

**Keywords:** retroperitoneal liposarcoma, well-differentiated liposarcoma, dedifferentiated liposarcoma, overall survival, prognostic factors, performance status, tumor size, real-world evidence

## Abstract

**Background/Objectives**: Histological subtype and resection completeness are established prognostic factors in retroperitoneal liposarcoma, but evidence often derives from resected primary disease. We characterized management and post-referral survival in a referral cohort. **Methods**: We retrospectively analyzed 61 patients managed from May 2015 to September 2025. Overall survival (OS) was measured from first presentation at our center. Kaplan–Meier and exploratory Cox analyses were performed, allowing the association between tumor diameter and mortality to vary over time. **Results**: Forty-two patients had primary disease, and 19 had recurrent disease; 12 had well-differentiated liposarcoma (WDLPS), and 49 had dedifferentiated liposarcoma (DDLPS). Among 24 patients who underwent surgery, 21 had macroscopically complete (R0/R1) resection. Twenty-five patients died. No deaths occurred in WDLPS; median OS was 29.8 months in DDLPS (*p* = 0.008). In DDLPS, recurrent versus primary disease was not associated with OS (hazard ratio [HR], 0.99; 95% confidence interval [CI], 0.41–2.38). Eastern Cooperative Oncology Group (ECOG) performance status ≥ 2 (n = 6 in the entire cohort) was associated with poorer OS (adjusted HR, 8.03; 95% CI, 2.20–29.36), and the association between tumor diameter and mortality varied over time (adjusted interaction HR, 1.44; 95% CI, 1.07–1.95). **Conclusions**: Observed post-referral survival differed by histological subtype. Among patients with DDLPS, no survival difference between primary and recurrent disease was demonstrated, but the wide CI and referral-related survivor selection preclude an inference of equivalence. Associations involving performance status and time-varying tumor diameter were exploratory and require external validation.

## Simple Summary

Retroperitoneal liposarcoma is a rare cancer whose behavior differs by histological subtype. We reviewed 61 patients with primary or recurrent disease managed at a specialized cancer center, including patients who did not undergo surgery. Among 24 surgically treated patients, macroscopically complete resection was achieved in 16 of 17 primary cases and five of seven recurrent cases. Among patients with dedifferentiated liposarcoma, no difference in survival after referral between primary and recurrent disease was demonstrated. This finding does not establish an equivalent prognosis because the sample was small and patients with recurrent disease had already survived from diagnosis to referral. Associations involving poor performance status and baseline tumor size were exploratory and imprecise. These findings support a cautious multidisciplinary assessment of patient condition, disease extent, tumor burden, resectability, and prior treatment history. Larger multicenter studies are needed.

## 1. Introduction

Retroperitoneal sarcoma (RPS) is a rare mesenchymal malignancy, with an estimated annual incidence of approximately 0.5–1 case per 100,000 population [1,2]. RPS comprises a heterogeneous group of tumors whose biological behavior, recurrence patterns, and treatment responses differ substantially across histological subtypes. Liposarcoma is the most common histological type of RPS, with well-differentiated liposarcoma (WDLPS) and dedifferentiated liposarcoma (DDLPS) being its predominant subtypes [1,2,3]. Owing to the large potential space in the retroperitoneum, these tumors may remain asymptomatic until they reach a considerable size. Moreover, their proximity to vital organs and major vessels makes macroscopically complete resection technically challenging and contributes to a substantial risk of local recurrence. Management by an experienced multidisciplinary team at a specialized sarcoma center is therefore recommended [1,2].

The WHO Classification recognizes WDLPS and DDLPS as distinct histological entities with different biological behavior [4]. WDLPS typically follows an indolent course and has little to no metastatic potential in the absence of dedifferentiation; nevertheless, repeated local recurrence can cause substantial morbidity and may ultimately become life-threatening [3,4]. By contrast, DDLPS is associated with a higher risk of both local and distant recurrence and greater disease-related mortality [3,4,5]. Histological subtype is therefore an established determinant of recurrence patterns and survival in patients with retroperitoneal liposarcoma [4,5,6,7]. Other clinical and pathological characteristics may also influence outcomes, including tumor grade and size, multifocality, completeness of resection, tumor rupture, patient age, and performance status [1,2,5,6,7].

For patients with localized and technically resectable primary RPS, macroscopically complete surgical resection remains the cornerstone of potentially curative treatment [1,2]. However, the roles of perioperative radiotherapy and systemic therapy remain incompletely defined. The phase III EORTC-62092 STRASS trial did not demonstrate an improvement in abdominal recurrence-free survival with preoperative radiotherapy in the overall study population, supporting a selective rather than routine approach to its use [8]. The management of recurrent, unresectable, or metastatic disease is even less standardized and generally requires individualized decisions in consideration of tumor biology, disease extent, previous treatments, expected morbidity, and patient preferences [1,2,9,10]. Much of the established prognostic evidence has been derived from patients who underwent resection for primary RPS [5,6,7]. Although these studies have provided essential information, their findings may not fully represent the broader population encountered in clinical practice, which includes patients presenting with recurrent disease and those managed using nonsurgical strategies [9,10,11].

In this study, we aimed to characterize patients with histologically confirmed retroperitoneal liposarcoma managed at a single specialized cancer center and to describe initial management strategies, resection status among surgically treated patients, and survival from the time of first presentation to our institution. The histological-subtype comparison was included to contextualize the cohort against established biological differences rather than to propose subtype as a novel prognostic factor. Among patients with DDLPS, we examined OS according to primary versus recurrent disease at presentation and explored clinical factors associated with OS.

## 2. Materials and Methods

### 2.1. Study Design and Patients

**Figure 1:**
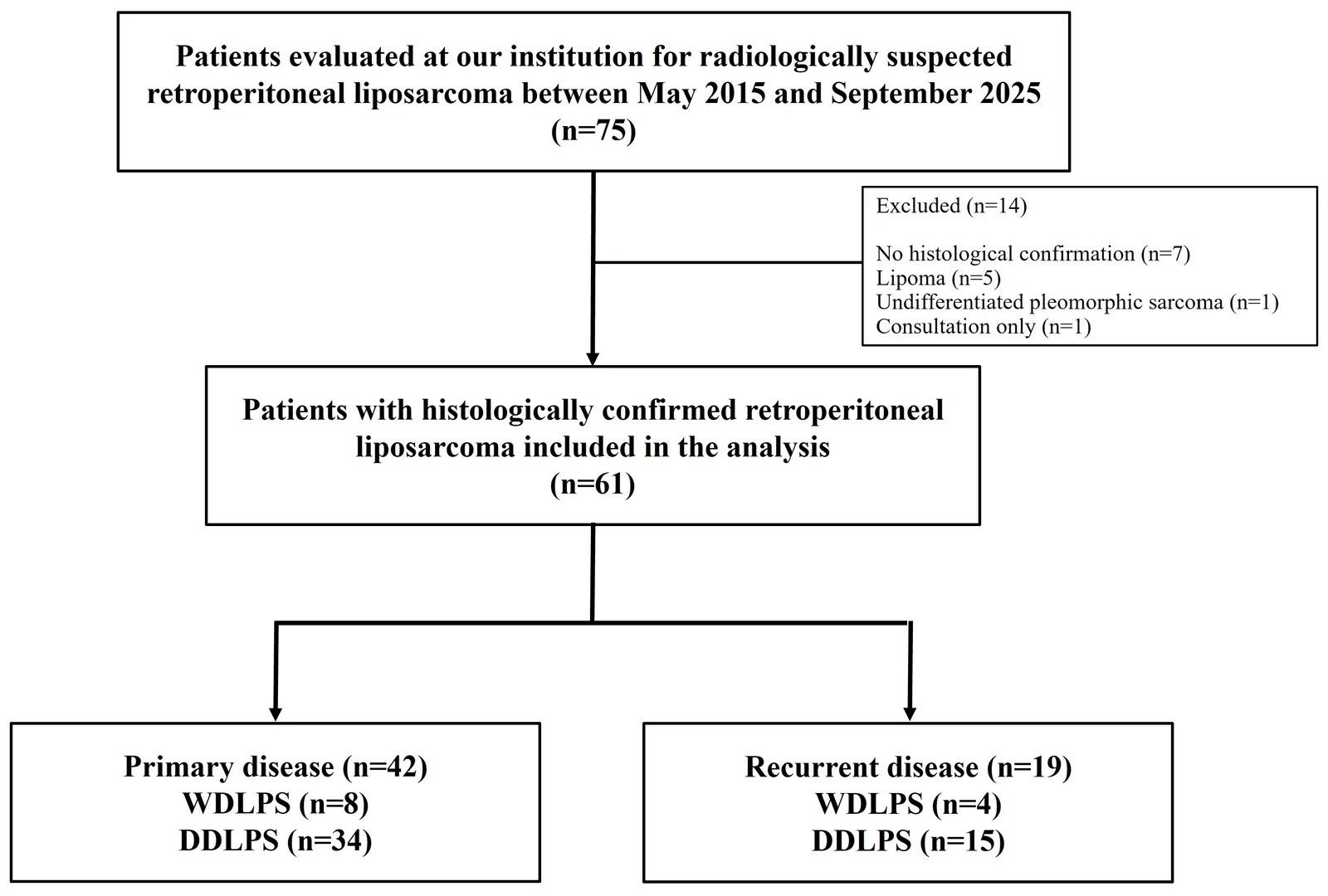
Patient selection and cohort composition. WDLPS, well-differentiated liposarcoma; DDLPS, dedifferentiated liposarcoma.

This single-center, retrospective cohort study included patients evaluated at the National Hospital Organization Kyushu Cancer Center between May 2015 and September 2025 for radiologically suspected retroperitoneal liposarcoma. Patients were eligible if the diagnosis of retroperitoneal liposarcoma was histologically confirmed using biopsy or resection specimens. Patients were excluded if histological confirmation was unavailable, the final pathological diagnosis was not liposarcoma, or they attended our institution for consultation only without subsequent longitudinal management. Both patients with untreated primary disease and those referred with recurrent disease after previous treatment were included. The patient selection process is shown in Figure 1.

No formal sample size calculation was performed owing to the rarity of retroperitoneal liposarcoma. All eligible patients identified during the study period were included.

### 2.2. Data Collection and Definitions

Clinical data were retrospectively extracted from electronic medical records, pathology reports, and radiological examinations. The variables collected at the first presentation to our institution included age, sex, Eastern Cooperative Oncology Group performance status (ECOG PS), disease status, histological subtype, maximum tumor diameter, presence of distant metastasis, and initial treatment strategy. Resection status and type of surgical procedure were additionally collected for patients who underwent surgery as part of the initial treatment at our institution. For patients presenting with recurrent disease, the interval from initial diagnosis to referral, whether referral occurred at the first or after multiple recurrences, and treatment modalities administered before referral were summarized. For patients managed without surgery at our institution, the principal reason for nonsurgical management was abstracted from the multidisciplinary treatment decision.

Primary disease was defined as retroperitoneal liposarcoma for which no previous disease-directed treatment had been administered before presentation to our institution. Recurrent disease was defined as locally recurrent or metastatic liposarcoma in patients who had received previous disease-directed treatment before referral to our institution. Accordingly, disease status represented each patient’s clinical status at the first presentation to our institution rather than the disease status at the initial diagnosis.

The maximum tumor diameter was defined as the longest documented diameter of the largest measurable retroperitoneal lesion on baseline computed tomography or magnetic resonance imaging available at the first presentation, before initiation of treatment at our institution. In patients with multifocal disease, the largest measurable lesion was used. Distant metastatic status was determined from the clinical and radiological assessment performed at presentation.

Histological diagnoses were based on biopsy or resection specimens evaluated either at our institution or at the referring institution. Pathological specimens obtained at referring institutions were not uniformly reviewed again at our institution; when institutional pathological review was not performed, the final pathology report issued by the referring institution was accepted. Histological subtype was classified as WDLPS or DDLPS according to the final documented pathological diagnosis. Ancillary diagnostic findings, including amplification analysis of the MDM2 proto-oncogene and immunohistochemistry for MDM2 and cyclin-dependent kinase 4 (CDK4), were incorporated into the pathological assessment when documented. Owing to the retrospective study design and inclusion of patients referred from other institutions, the methods used and availability of documented ancillary testing were not uniform across all patients.

### 2.3. Definition of Initial Treatment Strategies

The initial treatment strategy was defined as the first management strategy implemented at our institution after the initial assessment. Patients were assigned to one of six mutually exclusive categories: surgery alone, radiotherapy without planned surgery, preoperative radiotherapy followed by surgery, systemic therapy, observation, or best supportive care.

Treatment plans were determined at a multidisciplinary sarcoma conference attended principally by specialists in urology, diagnostic radiology, radiation oncology, medical oncology, orthopedic surgery, and gastrointestinal surgery, with other specialties participating as appropriate. When surgery was selected, the anticipated extent of resection was discussed before treatment. For patients managed without surgery, the principal reason was assigned to one of six mutually exclusive categories according to the main factor underlying the final multidisciplinary decision: locally advanced or unresectable disease; distant metastatic disease with prioritization of systemic or other nonsurgical treatment; poor general condition, comorbidity, or high anticipated operative risk; patient preference or refusal of surgery; observation for indolent or asymptomatic disease; or other reasons.

Surgery alone was defined as surgical resection without radiotherapy or systemic therapy incorporated into the initial treatment course. Radiotherapy without planned surgery was defined as radiotherapy delivered without a prospectively planned subsequent surgical resection. Preoperative radiotherapy followed by surgery was defined as radiotherapy administered with the intention to perform subsequent resection, followed by completion of the planned surgery. Systemic therapy was defined as initiation of anticancer drug therapy as the principal initial treatment strategy. Observation was defined as periodic clinical and radiological assessment without immediate disease-directed treatment. Best supportive care was defined as management focused on symptom relief and supportive measures without disease-directed anticancer treatment.

Treatments administered before referral in patients presenting with recurrent disease were recorded as previous treatment but were not used to determine the initial treatment category at our institution. Subsequent treatments administered after disease progression or a change in the treatment plan did not alter the initial treatment classification. Because treatment selection was individualized according to disease extent, resectability, patient condition, and clinical judgment, the present study was not designed to estimate or compare the causal effects of the different treatment strategies.

Among patients who underwent surgery as part of the initial treatment at our institution, procedures were classified descriptively as tumor resection alone or tumor resection with concomitant adjacent-organ resection. Resection status was abstracted from operative and pathology reports and classified as R0 (microscopically margin-negative), R1 (microscopically margin-positive without gross residual disease), or R2 (gross residual disease) [1,5]. Because microscopic margin status could not be consistently distinguished in the retrospective records, R0 and R1 were grouped as macroscopically complete resection for descriptive reporting. Resection status was classified as unknown when the available documentation was insufficient.

### 2.4. Outcome Measures and Follow-Up

The primary outcome was OS, calculated from the date of the first presentation at our institution to the date of death from any cause. Patients who were alive were censored on the date they were last known to be alive. For patients presenting with recurrent disease, survival time before their first presentation to our institution was not included in the OS analysis. The final data cutoff for vital status and follow-up was 8 September 2026.

Cause of death was determined from the available clinical records. Deaths documented as being attributable to retroperitoneal liposarcoma or its treatment were classified as disease-specific deaths. Follow-up duration was calculated from the first presentation to our institution and was estimated using the reverse Kaplan–Meier method [12].

### 2.5. Statistical Analysis

Continuous variables are presented as the median with an interquartile range, and categorical variables are presented as the number and percentage. Patient characteristics and initial treatment strategies were summarized descriptively, without formal statistical comparisons between the primary and recurrent disease groups. Resection status was summarized among patients who underwent surgery as part of the initial treatment at our institution; the corresponding denominators were 24 patients overall, 17 with primary disease, and 7 with recurrent disease.

OS was estimated using the Kaplan–Meier method, and median OS and survival probabilities at 1, 3, and 5 years were reported with 95% confidence intervals (CIs). Survival curves were compared using the log-rank test. OS was compared according to histological subtype in the entire cohort and according to disease status at presentation among patients with DDLPS.

Exploratory analyses of factors associated with OS were performed among patients with DDLPS using Cox regression models. The candidate variables were age, sex, ECOG PS, disease status at presentation, distant metastatic status, and maximum tumor diameter. Age was evaluated per 10-year increase, maximum tumor diameter per 5-cm increase, ECOG PS as ≥2 versus 0–1, disease status as recurrent versus primary disease, and distant metastatic status as present versus absent.

The proportional hazards assumption was assessed using Schoenfeld residuals [13]. Because the proportional hazards assumption was violated for maximum tumor diameter, tumor diameter was modeled using a time-varying coefficient by incorporating an interaction between maximum tumor diameter, expressed per 5-cm increase, and log(time/12) into the Cox model [13]. Time was expressed in months from the first presentation to our institution. The hazard ratio (HR) for a 5-cm increase in maximum tumor diameter at time *t* was therefore calculated as

*HR*(*t*) = exp{*β*_1_ + *β*_2_log(*t*/12)},

where *β*_1_ represents the main-effect coefficient for maximum tumor diameter, and *β*_2_ represents the coefficient for its interaction with log(time/12). Because log(12/12) = 0, the main-effect HR for maximum tumor diameter represents its association with mortality at 12 months. Model-based HRs for maximum tumor diameter were also estimated at 6, 12, 24, and 36 months.

The proportional hazards assumption was also violated for distant metastatic status. Therefore, distant metastatic status was incorporated as a stratification factor in the multivariable Cox model, allowing the baseline hazard to differ according to metastatic status; consequently, a single HR for distant metastasis was not reported. Given the limited number of outcome events, a parsimonious exploratory multivariable model was constructed that included ECOG PS and maximum tumor diameter, including its time-varying interaction term, and was stratified according to distant metastatic status. Covariate selection was based on clinical relevance, the number of observed events, univariable findings, and assessment of the proportional hazards assumption. Automated stepwise variable selection was not used.

There were no missing data for variables included in the survival analyses. Resection status was unknown for one surgically treated patient and was retained as an unknown category in the descriptive summary. All statistical tests were two-sided, and a *p* value < 0.05 was considered statistically significant. Because the analyses were exploratory, no adjustment was made for multiple comparisons. Statistical analyses were performed using EZR version 1.68 (Saitama Medical Center, Jichi Medical University, Saitama, Japan), a graphical user interface for R version 4.5.2 (R Foundation for Statistical Computing, Vienna, Austria) [14].

### 2.6. Ethical Considerations and Reporting

This study was conducted in accordance with the Declaration of Helsinki and was approved by the Institutional Review Board of the National Hospital Organization Kyushu Cancer Center (approval no. 2014-99; date of approval: 12 June 2024). The requirement for written informed consent to participate was waived because of the retrospective study design and use of existing clinical records. Information regarding the study was disclosed, and patients were provided with an opportunity to opt out. This study was reported in accordance with the Strengthening the Reporting of Observational Studies in Epidemiology (STROBE) statement [15].

## 3. Results

### 3.1. Patient Selection and Baseline Characteristics

A total of 75 patients were evaluated at our institution for radiologically suspected retroperitoneal liposarcoma between May 2015 and September 2025. Fourteen patients were excluded because of the absence of histological confirmation (n = 7), a final diagnosis of lipoma (n = 5) or undifferentiated pleomorphic sarcoma (n = 1), or attendance for consultation only without subsequent longitudinal management at our institution (n = 1). Consequently, 61 patients with histologically confirmed retroperitoneal liposarcoma were included in the analysis (Figure 1).

At presentation to our institution, 42 patients (68.9%) had primary disease and 19 (31.1%) had recurrent disease. Among patients with primary disease, eight had WDLPS and 34 had DDLPS; among patients with recurrent disease, four had WDLPS, and 15 had DDLPS. Overall, the cohort comprised 12 patients (19.7%) with WDLPS and 49 (80.3%) with DDLPS.

The baseline patient characteristics are summarized in Table 1. The median age at presentation was 67 years (interquartile range [IQR], 59–74 years), and 30 patients (49.2%) were male. ECOG PS was 0 in 38 patients (62.3%), 1 in 17 patients (27.9%), and ≥2 in six patients (9.8%). The median maximum tumor diameter was 12.0 cm (IQR, 7.5–18.0 cm), and 13 patients (21.3%) had distant metastases at presentation to our institution. The median maximum tumor diameter was 13.0 cm (IQR, 9.0–18.0 cm) among patients with primary disease and 8.0 cm (IQR, 4.8–15.5 cm) among those with recurrent disease. Distant metastases were present in 8 of 42 patients (19.0%) with primary disease and 5 of 19 patients (26.3%) with recurrent disease.

Among the 19 patients with recurrent disease (15 with DDLPS and 4 with WDLPS), the median interval from initial diagnosis to referral was 67.5 months (IQR, 33.6–140.8 months; range, 3.4–212.1 months). Ten patients (52.6%) were referred at their first recurrence and 9 (47.4%) after multiple recurrences. All 19 patients had undergone surgery before referral; 1 (5.3%) had received radiotherapy, and 2 (10.5%) had received systemic therapy before referral. Previous treatment modalities were not mutually exclusive.

### 3.2. Initial Treatment Strategies

The initial treatment strategies selected at our institution are shown in Table 1. Treatment involved surgery alone in 19 patients (31.1%), radiotherapy without planned surgery in seven patients (11.5%), and preoperative radiotherapy followed by surgery in five patients (8.2%). Systemic therapy was selected as the initial treatment strategy in 14 patients (23.0%). Nine patients (14.8%) underwent observation without immediate disease-directed treatment, and seven (11.5%) received best supportive care.

Among patients with primary disease, 12 (28.6%) underwent surgery alone, six (14.3%) received radiotherapy without planned surgery, and five patients (11.9%) received preoperative radiotherapy followed by surgery. Systemic therapy and observation were each selected in seven patients (16.7%), and best supportive care was selected in five (11.9%). Among patients presenting with recurrent disease, surgery alone and systemic therapy were each selected in seven patients (36.8%), whereas radiotherapy without planned surgery, observation, and best supportive care were selected in one (5.3%), two (10.5%), and two patients (10.5%), respectively. No patient with recurrent disease underwent preoperative radiotherapy followed by surgery as the initial treatment strategy at our institution.

Among the 37 patients managed without surgery at our institution, 25 had primary disease, and 12 had recurrent disease. In the primary-disease group, the principal reasons were locally advanced or unresectable disease in 10 patients (40.0%), poor general condition, comorbidity, or high anticipated operative risk in 6 (24.0%), distant metastatic disease with prioritization of systemic or other nonsurgical treatment in 4 (16.0%), observation for indolent or asymptomatic disease in 4 (16.0%), and prioritization of treatment for another active malignancy in 1 (4.0%). In the recurrent-disease group, the reasons were locally advanced or unresectable disease in 5 patients (41.7%), distant metastatic disease with prioritization of nonsurgical treatment in 5 (41.7%), and observation for indolent or asymptomatic disease in 2 (16.7%). Patient refusal was not the principal reason for nonsurgical management in any case.

Among the 24 patients who underwent surgery as part of the initial treatment at our institution, 7 (29.2%) underwent tumor resection alone and 17 (70.8%) underwent tumor resection with concomitant adjacent-organ resection. The corresponding counts were 5 and 12 among the 17 patients with primary disease and 2 and 5 among the 7 patients with recurrent disease, respectively. Overall, 21 patients (87.5%) underwent macroscopically complete resection (R0/R1), 2 (8.3%) had gross residual disease (R2), and resection status was unknown in 1 (4.2%). Among the patients with primary disease, 16 (94.1%) underwent macroscopically complete resection and 1 (5.9%) had gross residual disease. Among the patients with recurrent disease, 5 (71.4%) underwent macroscopically complete resection, 1 (14.3%) had gross residual disease, and resection status was unknown in 1 (14.3%) (Table 1).

### 3.3. Overall Survival (OS) in the Entire Cohort

At the data cutoff of 8 September 2026, the median follow-up duration for the entire cohort was 38.8 months (95% CI, 20.2–50.4 months). During follow-up, 25 patients (41.0%) died from any cause, including 22 disease-specific deaths (36.1%).

**Figure 2:**
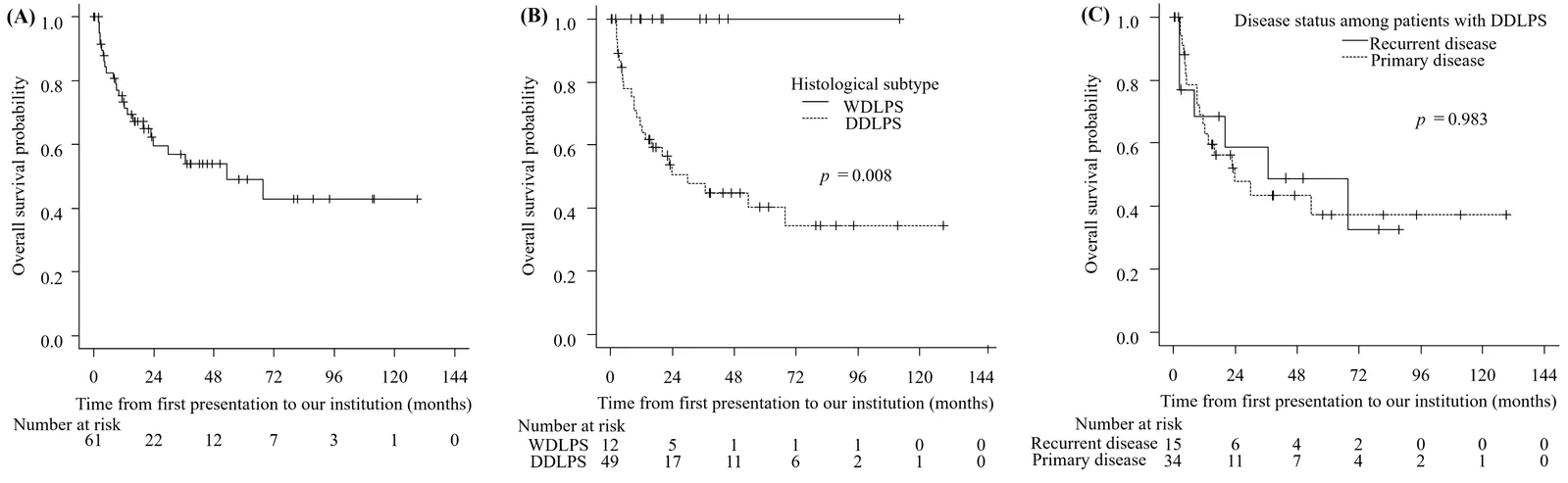
Kaplan–Meier estimates of overall survival. Overall survival was measured from the first presentation to our institution. (**A**) Overall survival in the entire cohort (n = 61). (**B**) Overall survival according to histological subtype: WDLPS (n = 12) versus DDLPS (n = 49). (**C**) Overall survival according to disease status at presentation among patients with DDLPS: primary disease (n = 34) versus recurrent disease (n = 15). WDLPS, well-differentiated liposarcoma; DDLPS, dedifferentiated liposarcoma.

The median OS for the entire cohort was 53.3 months (95% CI, 22.8 months–not reached). The estimated OS rates at 1, 3, and 5 years were 73.3% (95% CI, 59.6–83.0%), 56.8% (95% CI, 41.6–69.5%), and 49.1% (95% CI, 32.4–63.8%), respectively (Figure 2A and Table 2).

### 3.4. Overall Survival (OS) According to Histological Subtype

The median follow-up duration was 20.0 months (95% CI, 11.3–42.1 months) for patients with WDLPS and 50.4 months (95% CI, 23.2–79.9 months) for those with DDLPS. No deaths occurred among the 12 patients with WDLPS; therefore, median OS was not reached, and the estimated 1-, 3-, and 5-year OS rates were all 100%. The corresponding 95% CIs were not estimable because no death events occurred.

Among the 49 patients with DDLPS, 25 (51.0%) died from any cause, including 22 disease-specific deaths (44.9%). The median OS was 29.8 months (95% CI, 12.3 months–not reached). The estimated OS rates at 1, 3, and 5 years were 66.4% (95% CI, 50.5–78.2%), 47.8% (95% CI, 31.8–62.2%), and 40.3% (95% CI, 24.2–55.9%), respectively. OS differed significantly between the WDLPS and DDLPS groups (log-rank *p* = 0.008; Figure 2B and Table 2). A Cox regression HR for histological subtype was not estimated because no deaths occurred in the WDLPS group.

### 3.5. Overall Survival (OS) According to Disease Status Among Patients with DDLPS

Among the 49 patients with DDLPS, 34 had primary disease, and 15 had recurrent disease at presentation to our institution. OS did not differ significantly between patients presenting with recurrent disease and those presenting with primary disease (log-rank *p* = 0.983; Figure 2C). The corresponding univariable Cox model yielded an HR of 0.99 for recurrent versus primary disease (95% CI, 0.41–2.38; Table 3).

### 3.6. Exploratory Cox Regression Analyses Among Patients with DDLPS

The results of exploratory Cox regression analyses among patients with DDLPS are presented in Table 3. In the univariable analyses, ECOG PS ≥2 was associated with a higher risk of death than ECOG PS 0–1 (HR, 6.63; 95% CI, 2.28–19.26; *p* < 0.001). Age per 10-year increase (HR, 1.19; 95% CI, 0.85–1.66; *p* = 0.303), male sex (HR, 1.54; 95% CI, 0.70–3.39; *p* = 0.286), and recurrent disease at presentation (HR, 0.99; 95% CI, 0.41–2.38; *p* = 0.983) were not significantly associated with OS.

The proportional hazards assumption was violated for maximum tumor diameter (*p* = 0.032) and distant metastatic status (*p* = 0.027). Maximum tumor diameter was therefore modeled using a time-varying coefficient. In the univariable model, the HR per 5-cm increase in maximum tumor diameter at 12 months was 1.51 (95% CI, 1.14–2.00; *p* = 0.004), and the interaction between maximum tumor diameter and log(time/12) was significant (HR, 1.37; 95% CI, 1.02–1.84; *p* = 0.039). Because the proportional hazards assumption was violated for distant metastatic status, a single HR for distant metastasis was not determined.

In the multivariable model stratified according to distant metastatic status, ECOG PS ≥ 2 remained associated with a higher risk of death (adjusted HR, 8.03; 95% CI, 2.20–29.36; *p* = 0.002). The adjusted HR per 5-cm increase in maximum tumor diameter at 12 months was 1.34 (95% CI, 0.98–1.83; *p* = 0.066). The interaction between maximum tumor diameter and log(time/12) remained significant (adjusted HR, 1.44; 95% CI, 1.07–1.95; *p* = 0.016), indicating that the association between maximum tumor diameter and mortality varied over time.

The adjusted HRs for death per 5-cm increase in maximum tumor diameter were 1.04 (95% CI, 0.74–1.46) at 6 months, 1.34 (95% CI, 0.98–1.83) at 12 months, 1.73 (95% CI, 1.15–2.60) at 24 months, and 2.00 (95% CI, 1.22–3.29) at 36 months.

## 4. Discussion

In this single-center retrospective cohort of 61 patients with histologically confirmed retroperitoneal liposarcoma, the observed survival difference between WDLPS and DDLPS reproduced an established biological and prognostic distinction and is best viewed as a description of the case mix. The main finding requiring cautious interpretation was that no difference in post-referral OS between primary and recurrent DDLPS was demonstrated; the wide CI and referral-related survivor selection preclude an inference of equivalent prognosis. The associations of ECOG PS ≥ 2 and the time-varying effect of baseline maximum tumor diameter with mortality were strong but imprecise exploratory signals based on few patients and events, rather than validated independent prognostic factors. Resection status and procedure type described the center’s selected surgical practice and were not evaluated prognostically. Taken together, the results support multidimensional assessment at referral but do not establish new prognostic rules.

The survival difference between WDLPS and DDLPS is well established in the WHO Classification and previous studies [3,4,5,6,7,16,17,18]. WDLPS generally has an indolent course and minimal metastatic potential in the absence of dedifferentiation, although repeated local recurrence may cause substantial morbidity and can ultimately contribute to disease-related mortality. By contrast, DDLPS carries higher risks of local recurrence, distant metastasis, and death. Large multi-institutional studies have demonstrated that histological subtype is among the strongest determinants of recurrence pattern and long-term survival after resection of RPS [16,17,18]. In the present cohort, no deaths were observed among the 12 patients with WDLPS. This comparison was used to describe the case mix and confirm that the cohort reflected expected subtype-specific behavior, not to propose histological subtype as a novel prognostic factor. The median follow-up was shorter for WDLPS than for DDLPS, and few patients remained under observation at later time points; therefore, the absence of observed deaths should not be interpreted as evidence of negligible long-term mortality. Likewise, the overall estimated 5-year survival of 49.1% should not be compared directly with estimates from cohorts restricted to curative-intent resection for primary RPS because the present study also included recurrent or metastatic disease and nonsurgical management.

Among patients with DDLPS, no difference in post-referral OS between recurrent and primary disease was demonstrated (HR, 0.99; 95% CI, 0.41–2.38). Previous studies have shown that outcomes after recurrence of RPS are highly heterogeneous and are influenced by histological subtype, the pattern and number of recurrences, the disease-free interval, tumor growth rate, multifocality, resectability, and the feasibility of complete macroscopic resection [19,20,21,22,23]. The broader recurrent-disease group in this study was also heterogeneous: the interval from initial diagnosis to referral ranged from 3.4 to 212.1 months, 9 of 19 patients had experienced multiple recurrences, and all had undergone prior surgery, with small subsets also having received radiotherapy or systemic therapy. These descriptive data were available for all recurrent cases and were not used as covariates in the DDLPS-specific model. A binary classification of primary versus recurrent disease therefore cannot capture the underlying biological and clinical heterogeneity of DDLPS. More importantly, patients in the recurrent group had necessarily survived from their initial diagnosis and previous treatment until referral, whereas the primary group immediately included patients across the full spectrum of disease severity, including metastatic or unresectable disease and poor general condition. Because OS was measured from first presentation to our institution, this referral-related survivor selection may have attenuated a true difference between the groups. The wide CI around the HR remains compatible with clinically meaningful differences in either direction. Thus, the absence of a statistically significant difference does not establish equivalence; it indicates only that disease status at institutional presentation did not discriminate post-referral survival in this cohort.

ECOG PS ≥ 2 was associated with markedly poorer survival among patients with DDLPS, with an adjusted HR of 8.03. Performance status has also been associated with OS in patients with advanced WDLPS or DDLPS receiving systemic therapy [24]. ECOG PS may integrate tumor-related symptoms, disease burden, nutritional status, comorbidity, physiological reserve, and ability to undergo intensive treatment. Recent evidence also suggests that nutritional and body-composition measures may provide additional information in RPS: in a 250-patient surgical cohort, a low prognostic nutritional index was associated with severe postoperative complications and shorter OS, whereas the relationship between skeletal muscle index and OS depended on how it was modeled [25]. These indices were not systematically available in our cohort. Only six patients in the entire cohort had an ECOG PS of ≥2; the corresponding adjusted estimate had a wide CI, and the risk of model instability is therefore high. Poor performance status may also be correlated with tumor burden and may represent a consequence of advanced disease rather than an independent causal factor. Consequently, this result should be regarded as a strong but unstable exploratory signal, not validation of ECOG PS ≥ 2 as an independent prognostic factor or stand-alone threshold.

The time-varying association between maximum tumor diameter and mortality should likewise be considered a secondary exploratory finding. Tumor size has been incorporated into established prognostic models and associated with outcomes in several large RPS series [7,16,26], generally using a single, time-constant effect. In the present study, the proportional hazards assumption was violated for maximum tumor diameter; after modeling its interaction with log(time/12), the adjusted HR per 5-cm increase was 1.04 at 6 months, 1.34 at 12 months, 1.73 at 24 months, and 2.00 at 36 months. This approach more accurately represented the observed data than a single conventional Cox HR. Nevertheless, maximum diameter of the largest lesion is a rudimentary surrogate for disease burden. Its association with mortality may reflect total tumor volume, multifocality, anatomical involvement, surgical complexity, a lower probability of complete macroscopic resection, biological aggressiveness, or a greater likelihood of recurrence or progression. Only baseline diameter was evaluated; serial changes and growth rates were not analyzed, and the results do not indicate that individual tumors enlarged over time. The model was estimated from only 25 events while including ECOG PS, the main effect of diameter, its time interaction, and stratification by metastatic status; statistical stability is therefore limited. The temporal pattern remains hypothesis-generating and requires external validation.

The distribution of initial treatment strategies illustrates the clinical heterogeneity of the study population. Treatment plans were determined in a multidisciplinary sarcoma conference, and the most frequent reasons for nonsurgical management were locally advanced or unresectable disease, distant metastases prompting nonsurgical treatment, and poor general condition or operative risk. Complete macroscopic resection remains the cornerstone of potentially curative treatment for localized and technically resectable primary RPS [1,2,27,28]. However, the role of radiotherapy depends on the clinical context and histological subtype. The STRASS trial did not demonstrate an overall improvement in abdominal recurrence-free survival with preoperative radiotherapy [8], whereas the subsequent pooled analysis incorporating the off-trial STREXIT cohort suggested a possible benefit among selected patients with WDLPS or grade 1–2 DDLPS [29]. These findings, together with retrospective studies of perioperative radiotherapy, support selective use rather than a uniform approach [30,31]. Management of recurrent, metastatic, or unresectable DDLPS is even more individualized, and systemic treatment efficacy remains limited [23,24]. Treatment allocation in this cohort reflected resectability, disease extent, previous treatment, performance status, anticipated morbidity, and clinical judgment. The treatment groups therefore differed substantially in underlying prognosis, and the present data cannot support comparative effectiveness inferences. Radiotherapy without planned surgery and preoperative radiotherapy followed by surgery also represent clinically distinct strategies and should not be interpreted as a single radiotherapy group.

Completeness of resection is an established determinant of local control and survival in RPS [1,5,27,28]. Among the 24 surgically treated patients in this cohort, 7 underwent tumor resection alone, and 17 underwent concomitant adjacent-organ resection. Macroscopically complete R0/R1 resection was recorded in 16 of 17 primary cases and 5 of 7 recurrent cases; there were only 2 R2 resections overall, and status was unknown in 1 recurrent case. R0 and R1 were combined because microscopic margin status could not be distinguished reliably in the retrospective records. Given the small, highly selected surgical subgroup, the grouping of R0/R1, and the very small number of R2 cases, these data describe the center’s surgical practice and patient selection but do not permit prognostic inference regarding surgical radicality. Resection status was therefore not included in the full-cohort or DDLPS multivariable analyses.

From a clinical perspective, assessment at referral should extend beyond the distinction between primary and recurrent disease. Histological subtype, performance and nutritional status, disease distribution and tempo, tumor burden, prior treatment history, resectability, anticipated morbidity, and treatment goals should be considered together. This individualized approach is consistent with recommendations for multidisciplinary management at specialized sarcoma centers [1,23]. Treatment at low-volume institutions has been associated with inferior short- and long-term outcomes in population-based analyses, further supporting referral to centers with relevant expertise [32]. A strength of the present study is that all eligible patients with histologically confirmed retroperitoneal liposarcoma managed during the study period were included, including patients with primary and recurrent disease and those who did not undergo surgery. The cohort therefore complements literature derived predominantly from surgically treated primary RPS populations. Evaluation of the proportional hazards assumption and use of a time-varying coefficient also avoided imposing an unsupported constant effect for tumor diameter, although that analysis remains exploratory.

This study has several limitations, some of which have been discussed above. First, the retrospective, single-center design and small sample size limit generalizability and statistical precision. Only 25 deaths occurred, and only six patients had ECOG PS ≥ 2; despite use of a parsimonious model, the estimates remain susceptible to instability, residual confounding, and overfitting. Second, referral and selection biases were unavoidable, particularly among patients presenting with recurrent disease. Because survival before referral was not included, the findings describe prognosis from first presentation to our institution and should not be extrapolated to survival from initial diagnosis. Previous treatment, first versus multiple recurrence, and diagnosis-to-referral interval were summarized for the recurrent group but could not be incorporated into the DDLPS model. More detailed DDLPS-specific variables—including FNCLCC grade, the extent of the dedifferentiated component, multifocality, detailed anatomical involvement, disease-free interval, and the exact number and pattern of previous recurrences and resections—were not uniformly available. Third, pathological specimens obtained at referring institutions were not uniformly re-reviewed, and ancillary testing for MDM2 and CDK4 was not uniform; some pathological misclassification cannot be excluded. Fourth, maximum tumor diameter was a single measurement of the largest lesion and did not capture total tumor volume, multifocality, anatomical involvement, or subsequent kinetics. Fifth, prognostic nutritional index and CT-derived body-composition measures were not systematically collected. Sixth, resection status was available only for 24 selected surgical patients; R0 and R1 could not be separated reliably, and the established prognostic influence of microscopic margin status could not be evaluated. Seventh, treatment strategies were heterogeneous and strongly confounded by indication, so no causal treatment comparisons were attempted. Finally, the time-dependent tumor-diameter analysis was exploratory; no adjustment was made for multiple testing, and all prognostic findings require validation in larger, preferably multicenter cohorts.

## 5. Conclusions

Observed post-referral survival patterns differed according to histological subtype, although the absence of deaths among 12 patients with WDLPS should be viewed as descriptive. Among patients with DDLPS, no difference in post-referral OS between primary and recurrent disease was demonstrated; this finding does not establish equivalence because of limited precision and referral-related survivor selection. Associations involving ECOG PS and the time-varying effect of baseline tumor diameter were exploratory and require validation. Resection status described a selected surgical subgroup and should not be interpreted prognostically. The substantial mortality observed among patients with DDLPS highlights the need for coordinated multidisciplinary, patient-centered care and continued development of more effective local and systemic treatment strategies.

## Figures and Tables

**Table 1:** Baseline characteristics, initial treatment strategies, and resection status according to disease status at presentation.

Characteristic	Total (n = 61)	Primary Disease (n = 42)	Recurrent Disease (n = 19)
Age at presentation (years), median (IQR)	67 (59–74)	70 (61–75)	64 (58–72)
Male sex, n (%)	30 (49.2)	23 (54.8)	7 (36.8)
ECOG performance status, n (%)			
0	38 (62.3)	26 (61.9)	12 (63.2)
1	17 (27.9)	13 (31.0)	4 (21.1)
≥2	6 (9.8)	3 (7.1)	3 (15.8)
Histological subtype, n (%)			
WDLPS	12 (19.7)	8 (19.0)	4 (21.1)
DDLPS	49 (80.3)	34 (81.0)	15 (78.9)
Maximum tumor diameter (cm), median (IQR)	12.0 (7.5–18.0)	13.0 (9.0–18.0)	8.0 (4.8–15.5)
Distant metastasis at presentation to our institution, n (%)	13 (21.3)	8 (19.0)	5 (26.3)
Initial treatment strategy at our institution, n (%)			
Surgery alone	19 (31.1)	12 (28.6)	7 (36.8)
Radiotherapy without planned surgery	7 (11.5)	6 (14.3)	1 (5.3)
Preoperative radiotherapy followed by surgery	5 (8.2)	5 (11.9)	0 (0.0)
Systemic therapy	14 (23.0)	7 (16.7)	7 (36.8)
Observation	9 (14.8)	7 (16.7)	2 (10.5)
Best supportive care	7 (11.5)	5 (11.9)	2 (10.5)
Resection status among surgically treated patients, n (%)			
R0/R1 (macroscopically complete)	21 (87.5)	16 (94.1)	5 (71.4)
R2 (gross residual disease)	2 (8.3)	1 (5.9)	1 (14.3)
Unknown	1 (4.2)	0 (0.0)	1 (14.3)

Data are presented as n (%) unless otherwise indicated; continuous variables are presented as medians (IQR). Percentages were calculated within columns, except for resection status, for which the denominators were patients who underwent surgery as part of the initial treatment at our institution (total, n = 24; primary disease, n = 17; recurrent disease, n = 7). R0 and R1 were grouped as macroscopically complete resections because microscopic margin status could not be consistently distinguished; R2 indicates gross residual disease. No formal between-group comparisons were made. DDLPS, dedifferentiated liposarcoma; ECOG, Eastern Cooperative Oncology Group; IQR, interquartile range; R0, microscopically margin-negative resection; R1, microscopically margin-positive resection without gross residual disease; R2, gross residual disease; WDLPS, well-differentiated liposarcoma.

**Table 2:** Overall survival outcomes in the entire cohort and according to histological subtype.

Variable	Overall (n = 61)	WDLPS (n = 12)	DDLPS (n = 49)
Median follow-up, months (95% CI)	38.8 (20.2–50.4)	20.0 (11.3–42.1)	50.4 (23.2–79.9)
Death from any cause, n (%)	25 (41.0)	0 (0)	25 (51.0)
Disease-specific death, n (%)	22 (36.1)	0 (0)	22 (44.9)
Median OS, months (95% CI)	53.3 (22.8–NR)	NR	29.8 (12.3–NR)
1-year OS, % (95% CI)	73.3 (59.6–83.0)	100 (NE)	66.4 (50.5–78.2)
3-year OS, % (95% CI)	56.8 (41.6–69.5)	100 (NE)	47.8 (31.8–62.2)
5-year OS, % (95% CI)	49.1 (32.4–63.8)	100 (NE)	40.3 (24.2–55.9)

Data are presented as n (%) unless otherwise indicated. Follow-up was estimated using the reverse Kaplan–Meier method, and OS was estimated from first presentation at our institution to death from any cause; patients alive at last follow-up were censored. OS differed between the WDLPS and DDLPS groups (log-rank *p* = 0.008). Because no deaths occurred in the WDLPS group, median OS was not reached, and the 95% CIs for the 1-, 3-, and 5-year OS rates were not estimable. CI, confidence interval; DDLPS, dedifferentiated liposarcoma; NE, not estimable; NR, not reached; OS, overall survival; WDLPS, well-differentiated liposarcoma.

**Table 3:** Exploratory Cox regression analyses of factors associated with overall survival among patients with dedifferentiated liposarcoma.

Variable	Comparison/Unit	Univariable HR (95% CI)	*p* Value	Multivariable HR (95% CI)	*p* Value
Age	Per 10-year increase	1.19 (0.85–1.66)	0.303	—	—
Sex	Male vs. female	1.54 (0.70–3.39)	0.286	—	—
ECOG PS	≥2 vs. 0–1	6.63 (2.28–19.26)	<0.001	8.03 (2.20–29.36)	0.002
Disease status at presentation	Recurrent vs. primary	0.99 (0.41–2.38)	0.983	—	—
Distant metastasis at presentation ^1^	Present vs. absent	—	—	Stratification factor	—
Maximum tumor diameter ^2^	Per 5-cm increase at 12 months	1.51 (1.14–2.00)	0.004	1.34 (0.98–1.83)	0.066
Maximum tumor diameter × log(time/12) ^2^	Time-varying interaction term	1.37 (1.02–1.84)	0.039	1.44 (1.07–1.95)	0.016

HRs for age and maximum tumor diameter are expressed per 10-year and 5-cm increases, respectively. ^1^ Because distant metastatic status violated the proportional hazards assumption, it was included as a stratification factor in the multivariable model; therefore, no single HR was estimated. ^2^ Maximum tumor diameter was modeled using an interaction with log(time/12); the main-effect HR represents the effect at 12 months. The multivariable model included ECOG PS, maximum tumor diameter, and the time-varying interaction, with stratification by distant metastatic status. All *p* values are two-sided, with no adjustment for multiple comparisons. CI, confidence interval; ECOG PS, Eastern Cooperative Oncology Group performance status; HR, hazard ratio.

## Data Availability

The datasets generated and/or analyzed during the current study are not publicly available because they contain potentially identifiable clinical information but are available from the corresponding author on reasonable request, subject to institutional and ethical approval.
